# Charting exposomethics: a roadmap for the ethical foundations of the human exposome project

**DOI:** 10.1186/s40246-025-00906-7

**Published:** 2026-01-17

**Authors:** Fenna C. M. Sillé, Myriem Belkadi, Kirsten Koehler, Joseph Ali, Vasilis Vasiliou, Denis Sarigiannis, Thomas Hartung

**Affiliations:** 1 Center for Alternatives to Animal Testing (CAAT), Department of Environmental Health & Engineering, Johns Hopkins Bloomberg School of Public Health and Whiting School of Engineering, Johns Hopkins University, Baltimore, MD 21205 USA; 2 Department of Environmental Health & Engineering, Johns Hopkins Bloomberg School of Public Health and Whiting School of Engineering, Johns Hopkins University, Baltimore, MD 21205 USA; 3 Johns Hopkins Berman Institute of Bioethics, Johns Hopkins University, Baltimore, MD 21205 USA; 4 Department of International Health, Johns Hopkins Bloomberg School of Public Health, Johns Hopkins University, Baltimore, MD 21205 USA; 5 Department of Environmental Health Sciences, Yale School of Public Health, New Haven, CT 06510 USA; 6 National Hellenic Research Foundation, 48 Vassileos Constantinou Avenue, 11635 Athens, Greece; 7 Department of Chemical Engineering, Environmental Engineering Laboratory, Aristotle University of Thessaloniki, University Campus, 54124 Thessaloniki, Greece; 8 HERACLES Research Center on the Exposome and Health, Center for Interdisciplinary Research and Innovation, Aristotle University of Thessaloniki, Balkan Center, 57001 Thessaloniki, Greece; 9 School of Medicine, University School for Advanced Study (IUSS), Science, Technology and Society Department, Environmental Health Engineering, 27100 Pavia, Italy; 10 Doerenkamp-Zbinden-Chair for Evidence-Based Toxicology, Department of Environmental Health & Engineering, Johns Hopkins Bloomberg School of Public Health and Whiting School of Engineering, Johns Hopkins University, Baltimore, MD 21205 USA; 11 CAAT-Europe, University of Konstanz, Universitätsstraße 10, 78464 Constance, Germany

## Abstract

**Background:**

The Human Exposome Project (HEP) aims to chart lifelong environmental exposures and their biological consequences, furnishing the environmental counterpart to the genomic revolution. Yet the fine‑grained, multimodal data streams that fuel exposomics—biospecimens, geolocation traces, wearable‑sensor feeds, and socio‑environmental metadata—raise privacy, justice, and governance questions that may exceed the reach of conventional bioethics.

**Main body:**

Building on lessons from genomics, biobanking, digital health, and environmental‑justice research, we identify five foundational ethical domains for exposome science: (1) privacy and data sovereignty, (2) informed consent and sustained participant engagement, (3) environmental justice, (4) governance and oversight, and (5) actionability and the responsible return of results,as well as (6)the adherence to research program goals. Similar to the “values in design” construct widely used in the socio-technical field and the “ethics by design” in the artificial intelligence (AI) field, we translate these domains into operational pillars for ethics‑by‑design research practice: dynamic or tiered consent architectures; participatory governance mechanisms such as community advisory boards; embedded ethics research programs; algorithmic‑fairness protocols for artificial‑intelligence analytics; and dedicated review bodies equipped to evaluate longitudinal, sensor‑based, multi‑omics studies. Concrete recommendations include federated data stewardship to minimize re‑identification risk, Evidence‑to‑Decision frameworks that couple exposomic evidence with societal values, and transparent pathways for communicating context‑dependent findings to individuals, communities, and policymakers.

**Conclusions:**

Ethical preparedness and action are a prerequisite for the scientific impact and social license of exposome research. Institutionalizing the proposed roadmap—via an international Exposome Ethics Consortium, expanded training for Institutional Review Boards, harmonized regulatory guidance, and sustained community co‑governance—will help protect privacy, promote equity, and foster public trust. Embedding systematic ethical reflection as core infrastructure will enable the Human Exposome Project to realize its promise of precision public health without replicating patterns of opaque surveillance, marginalization, or data commodification.

**Plain Language Summary:**

The Human Exposome Project (HEP) represents an ambitious endeavor to characterize lifelong environmental exposures in relation to health. Yet, this vision brings profound ethical challenges: from managing massive, sensitive datasets to ensuring justice for disproportionately exposed communities. This article synthesizes foundational work on exposome ethics, outlines core ethical challenges, and proposes a proactive ethical governance model that ensures scientific integrity and social legitimacy.

## Background: why ethics for the human exposome project?

The Human Exposome Project (HEP) represents a transformative vision for environmental health research: to comprehensively map the totality of environmental exposures (chemical, physical, biological, and psycho-social) from conception to death and their cumulative effects on human biology [1]. Coined by Christopher Wild (2005) [2], the exposome complements the genome by addressing the far more variable and less understood environmental contributors to disease, which account for an estimated 70–90% of disease risks [3]. Building on this vision, large-scale exposome research programs, such as the European Human Exposome Network (EHEN),^1^ HHEAR^2^ (US), or ATHLETE,^3^ aim to integrate sensor-based monitoring, biobanking, and high-throughput-omics to track exposures and their biological responses across time [4, 5]. As AI-enabled multi-omics approaches become foundational to precision public health, the exposome now mirrors the transformative moment once marked by the Human Genome Project, calling for an ethical framework that is anticipatory, scalable, and socially grounded. Most recently, we convened a Human Exposome Moonshot Forum^4^ to evaluate the feasibility and willingness of research and civil society to embark on a Human Exposome Project^5^ [6].

It is important to clarify that the HEP referenced throughout this manuscript is, at present, a visionary and coordinated international proposal, analogous in scale and ambition to the Human Genome Project, rather than a single, funded, and currently underway initiative. Notably, together the fragmented exposomics research has received several hundred million $ of funding, especially in the EU, US, and China, but a coordinated global project is only emerging. Recent efforts, such as the Human Exposome Moonshot Forum^6^ we convened [7], are actively building the scientific consensus and institutional momentum necessary to launch such an endeavor and to evaluate the feasibility and willingness of research and the civil society to embark on. This manuscript's purpose is to provide the critical ethical roadmap required before such a project is fully realized, ensuring that ethical considerations are embedded in its design from the very beginning, rather than being reactive additions.

However, This ambitious scientific project raises profound ethical challenges that distinguish it from genomics, traditional epidemiology, and even environmental health science. Exposome research operates through continuous, ideally passive, data collection that includes biospecimens, location data, wearable sensor feeds, and environmental sampling, generating highly granular and potentially re-identifiable information [8]. The ethical implications of such work span consent, privacy, data ownership, and the fair inclusion and benefit-sharing with disproportionately burdened populations, often without fully articulated normative frameworks to guide decision-making [9, 10].

It is crucial to distinguish the proposed HEP from the Human Epigenome Project, as they represent fundamentally different scientific and ethical domains. The Human Epigenome Project is a well-established initiative focused on mapping specific, heritable chemical modifications—primarily DNA methylation patterns, histone modifications, and small non-coding RNAs [11]—that regulate gene expression without altering the underlying DNA sequence [12, 13]. Its goal is to create reference profiles of these "epigenetic marks" to understand their role in development, cell specialization, and disease, particularly cancer. In essence, it deciphers the biological software that instructs cells on how to use the genomic hardware. The fundamental distinction lies in their primary subjects: the Epigenome Project investigates the molecular mechanisms of gene regulation, whereas the Exposome Project investigates the external and internal environments that influence health and trigger those molecular mechanisms. This difference in scale and focus leads to distinct ethical challenges [14, 15]; while epigenomics raises concerns about genetic-like discrimination and data privacy, exposomics introduces unprecedented issues related to continuous surveillance, data sovereignty of communal environmental information, and justice in addressing societal-level exposure sources.

Unlike the genomics field, where early investment in ethical inquiry led to the creation of the Ethical, Legal, and Social Implications (ELSI)^7^ program, exposome ethics remains underdeveloped, fragmented, and largely reactive [16]. The exposome’s deeply interwoven biological and environmental data fundamentally challenge the individualistic traditions that have often shaped ethical reasoning within genomics [17, 18]. Unlike the genome, which is contained within an individual body, the exposome is inherently relational and collective: an individual’s biomarker data is a point-in-time reflection of community-level environmental conditions, social policies, and economic structures. While lessons can be drawn from the ELSI framework, its ethical component historically emphasized individual autonomy and privacy, whereas the legal and social strands have increasingly addressed collective and intergenerational concerns. Exposome ethics builds directly on these collective dimensions, seeking to integrate them as core design principles rather than adjuncts. Accordingly, an exposome-ethics framework must transcend individual protection alone by prioritizing collective rights, participatory governance, and the political capacity for communities to act on findings. Ethical preparedness therefore entails not only safeguarding participants but also enabling communities to convert exposomic evidence into social and regulatory change, i.e., a shift from protection to empowerment and structural justice.

This article aims to address that gap. Building on systematic reviews, emerging governance frameworks, and participatory models, we propose a structured ethical roadmap for the HEP. This includes six core domains: privacy and data sovereignty, informed consent and engagement, environmental justice, ethical and adaptive governance, the actionable interpretation of findings and the adherence to research program goals, i.e. the ethical alignment of exposome research objectives with public and planetary health needs. By foregrounding these ethical considerations, we seek to ensure that the HEP not only advances scientific knowledge but also promotes equity, trust, and democratic accountability. As exposomics advances toward predictive, AI-driven approaches in population health, the success of such initiatives rests not only on scientific progress but also on adherence to ethical standards aligned with public health objectives and environmental justice. To address these requirements, we incorporate a robust ethics framework encompassing objectives, protocols, tools, and governance within the scientific architecture of the HEP.

## Learning from precedents

The ethical challenges posed by exposome research are not entirely unprecedented. Valuable lessons can be drawn from adjacent fields that have grappled with similar issues, particularly genomics, biobanking, and environmental health. Each domain offers important insights and cautionary tales into how ethical foresight, or the lack thereof, can shape public trust, policy development, and the long-term sustainability of scientific programs.

### Genomics and the ELSI legacy: a foundation and a caution

The HGP established the ELSI program as a proactive effort to address the normative ramifications of large-scale genetic data collection and use. ELSI succeeded in mainstreaming discussions of informed consent, genetic discrimination, and the return of individual research results. It is important to acknowledge that the ELSI field itself has evolved considerably and has been instrumental in advancing more adaptive approaches, such as dynamic consent [19] and participatory engagement.

However, the structural and normative legacy of the early genomic era, upon which many current governance frameworks are built, had inherent limitations when applied to the exposome, most notably, its predominantly individualistic framing of consent, its difficulty in anticipating future data uses, and its limited mechanisms for sustained participant engagement [16, 20]. This legacy often includes a primary focus on the individual as the unit of ethical concern, models of consent designed for more discrete and predictable data types, and frameworks that are less equipped to address community-level data and harms. These limitations, which contemporary ELSI scholarship actively seeks to overcome, are particularly salient for exposome science.

Therefore, exposome ethics must build directly upon the progressive advancements within ELSI research. The exposome’s inherently multi-modal, longitudinal, and community-embedded nature demands ethical infrastructures that are participatory and justice-oriented by design. The proposed framework for the HEP aims to integrate and extend these modern ELSI principles, tailoring them to the specific sociotechnical complexities of environmental exposure data.

These same limitations pose risks for exposome science. Consent in exposomics is further complicated by its untargeted, discovery-driven nature and the potential for incidental findings that extend far beyond the original research intent. As Safarlou et al. argue, the traditional model of informed consent, predicated on discrete decisions and well-defined risks, is fundamentally ill-suited to exposome research. Instead, what is required is a shift toward ongoing, action-oriented models of participation that can better accommodate uncertainty and evolving risk–benefit profiles [21].

Building on these insights, exposome ethics must critically advance beyond the genomics paradigm (Table 1). Notably, several Exposome projects included ethics components (Table 2).

The ELSI program’s fundamental limitation for exposomics is its historical focus on the individual as the main unit of ethical concern - protecting autonomy, privacy, and preventing genetic discrimination. While indispensable, this framing is insufficient for the exposome. Exposome data, by its nature, link the individual to the community and the environment; a pollutant level in one person’s blood is a marker of a shared environmental reality. Accordingly, the ethical questions expand from “*How do we protect this person’s data?*” to “*Who benefits from this knowledge? Who decides the research agenda? How are responsibility and power distributed across communities, governments, and institutions to act on the evidence generated?*” Exposome ethics must recognize that affected communities often lack the structural power to implement change; therefore, governments, funders, and regulatory bodies share an affirmative obligation to translate exposomic findings into policy and remediation.

Exposomics, with its inherently multi-modal, longitudinal, and community-embedded nature, calls for ethical infrastructures that are participatory by design. The proposed Exposome Ethics Consortium builds on the spirit of ELSI but is tailored to the sociotechnical complexity of exposome science, emphasizing dynamic consent, data sovereignty, and justice-informed governance as essential pillars of legitimacy and public trust. The limitations of the ELSI model underscore the importance of developing exposome-specific ethical infrastructures grounded in inclusivity, dynamic participation, and ongoing deliberation. We propose expanding these lessons to address six foundational domains relevant to exposome ethics.

### Biobanking: storage, reuse, and the ethics of temporal drift

Biobanks offer another instructive precedent. Initially envisioned as repositories for specific studies, biobanks have evolved into dynamic infrastructures supporting secondary research, population monitoring, and precision medicine. This evolution, however, has exposed tensions between the long-term utility of stored biospecimens and the ethical foundations of their collection. Issues of re-consent, commercialization, data linkage, and control over downstream uses have led to public controversies, legal challenges, and declining participation in some settings [22, 23].

These issues are magnified in the exposome context. Exposome biobanking often requires storing and integrating data from multiple life stages, including prenatal and pediatric samples, with longitudinal behavioral and environmental data. Moreover, the potential future uses of these datasets, including machine learning, digital epidemiology, urban planning, or integration with socio-economic profiles, and the impact of future results are difficult to fully anticipate at the time of collection. Consequently, exposome biobanks must adopt governance models that combine robust stewardship, transparency, and participant agency over time [8, 9].

### Environmental health and justice: lessons in trust and disparity

Environmental health research has a long history of engagement with marginalized communities, often under conditions of profound distrust, resource inequality, and environmental racism. Communities most affected by pollution, industrial activity, and poor infrastructure have also been those least empowered to influence research agendas or benefit from resulting interventions [24].

Exposome research risks repeating these dynamics if ethical frameworks fail to address structural power imbalances. The data-intensive nature of exposomics may render low-income and racialized communities hypervisible to state, academic, or commercial actors—without commensurate protections or participatory influence. Exposome researchers must therefore prioritize environmental justice not as an optional add-on, but as a central principle guiding data collection, study design, and benefit-sharing strategies. Participatory models such as community-based participatory research (CBPR) and “*citizen science with power*” offer promising avenues for ethical alignment, but require institutional commitment and funding to ensure that researchers can meet the expectations established between the community and the scientists in the preliminary stages of the research [25, 26].

## Key ethical dimensions of exposome research

**Fig. 1:**
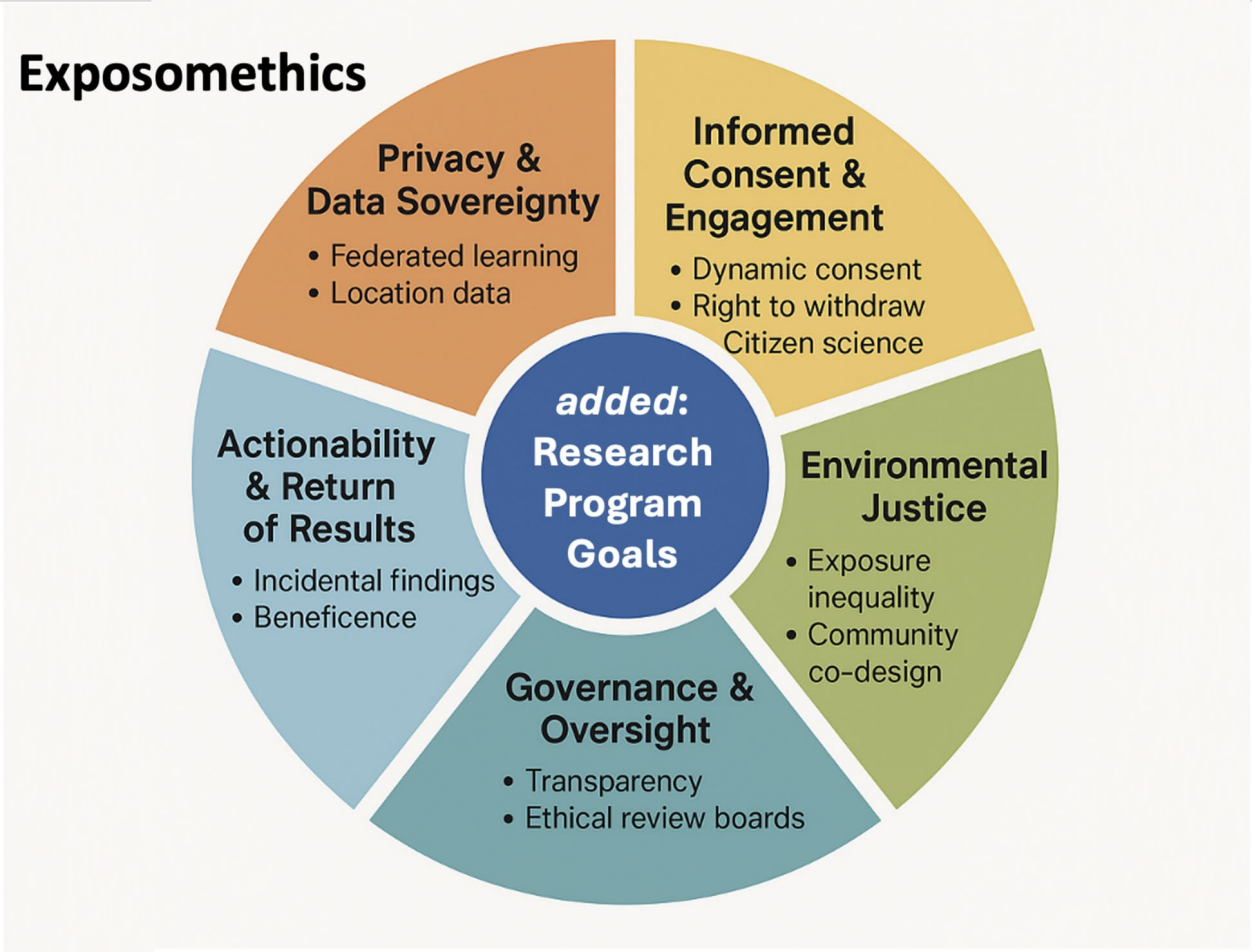
Ethical dimensions of the human exposome project. A conceptual wheel showing five core domains: privacy and data sovereignty, informed consent and engagement, environmental justice, governance and oversight, and actionability and return of results. Each domain includes 2–3 subpoints illustrating key concerns

**Fig. 2:**
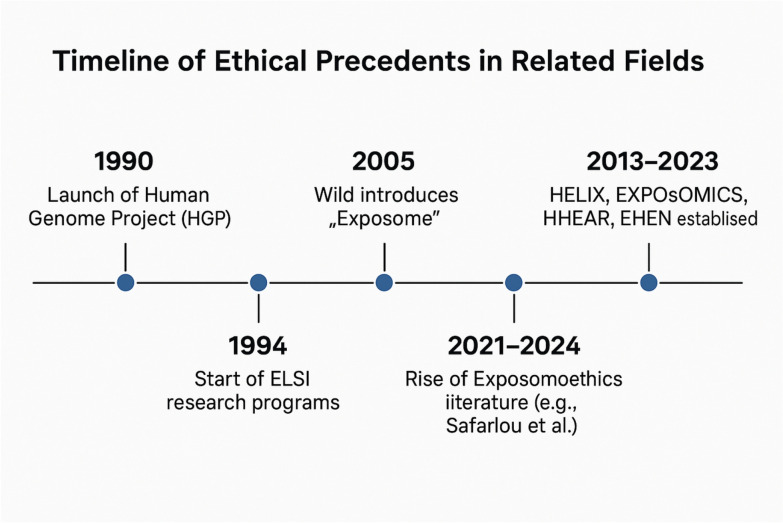
Timeline of ethical precedents in related fields. A horizontal timeline illustrating milestones such as the Human Genome Project (1990), introduction of ELSI (1994), exposome concept (2005), and major exposome initiatives (2013–2024)

The ethical complexity of HEP stems not only from its technical ambition, but from the sociotechnical entanglements it creates. As exposome science blurs the lines between individual biology and environmental context, five key ethical dimensions have emerged as pillars of responsible practice (Fig. 1). These include (1) privacy and data sovereignty, (2) informed consent and participant engagement, (3) equity and environmental justice, (4) governance and oversight, and (5) the actionability of results. Each of these dimensions intersects with the others and must be considered in the design and governance of exposome research infrastructures. A timeline of important developments is given in Fig. 2. Herein, we expand Safarlou et al.’s five ethical themes model to incorporate an additional, foundational ethical domain, i.e., the adherence to research program goals, reflecting the ethical necessity of aligning exposome science with pressing public health priorities. This expanded model (Box 1) guides the following analysis.

**Box 1:** The six ethical domains in exposome research

Safarlou et al. [8] propose a systematic ethical framework composed of five domains, here expanding and adding a sixth:
1. *Privacy and Data Sovereignty*—Exposome data are dynamic, highly sensitive, and often re-identifiable even when anonymized, demanding privacy anchored in individual/community data sovereignty and context-aware governance, complemented—not replaced—by safeguards like federated learning and encryption.
2. *Research Standards*—Assurance of methodological rigor, data quality, and transparency in multidisciplinary exposomics.
3. *Research Tools*—Evaluation of the ethical implications of sensors, AI algorithms, biobanking, and data platforms used in exposome science.
4. *Study Participants*—Protection of autonomy, informed consent, and justice, especially in vulnerable populations across life stages.
5. *Consequences of Research Products*—Attention to how results are interpreted, used in policy, and whether they advance or inhibit social justice.
This domain model helps identify ethical blind spots and supports the rationale for establishing a dedicated *'Exposome Ethics Consortium'* to anticipate and coordinate governance in this evolving field.
6. *Research Program Goals*—Ethical alignment of exposome research objectives with public and planetary health needs, prioritizing justice-driven and non-extractive research designs. This sixth dimension expands on Safarlou et al.'s five ethical domains.

The following explicit definitions shall clarify these core concepts:

"*Ethics-by-Design*" refers to the integration of ethical analysis, particularly concerning justice, equity, and power, into every stage of the research lifecycle, from the technical design of data platforms to the planning of stakeholder engagement.

"*Data Sovereignty*" in the exposome context extends beyond individual control to encompass community sovereignty—the right of a community to govern the collection, ownership, and application of data about their shared environment and health.

"*Dynamic Consent*” is a process that supports ongoing communication and choice, allowing participants and communities to re-negotiate or withdraw consent as research evolves. It provides mechanisms for individuals to modify or revoke permission for future data uses, and for communities—through agreed governance bodies such as Community Advisory Boards—to collectively decide whether continued participation aligns with their interests. This reflects the longitudinal and uncertain nature of exposome studies and embeds both individual autonomy and collective agency in consent practice.

### Privacy and data sovereignty

Exposome research generates highly sensitive, multi-dimensional data-including biological samples, behavioral patterns, geographic location, and longitudinal environmental exposures that can reveal intimate details about an individual’s health status, social context, and life history. Even when anonymized, exposome data often contain quasi-identifiers (e.g., time-stamped GPS tracks, household air quality, or metabolomic fingerprints) that can enable re-identification when cross-linked with public or commercial databases [16, 27].

As Safarlou et al. [16] argue, privacy in exposomics cannot be reduced to intrinsic data characteristics alone but must instead be grounded in a normative understanding of what data reveals about a person’s life and autonomy. This perspective calls for evaluating privacy not merely through technical safeguards, but through ongoing reflection on context, use, and potential social harms. It positions data sensitivity as inherently social and value-laden, especially in the context of environmental surveillance, geolocation tracking, and exposure profiling.

Unlike genetic data, which is often seen as static and immutable, exposome data is dynamic and reflective of temporally evolving behaviors and exposures. The principle of data sovereignty, i.e., the right of individuals and communities to control the use of their data, is critically important for exposome research because the data itself is inherently communal and highly re-identifiable. Unlike a genetic sequence, which is primarily personal, a GPS track or local air quality measurement reveals information about a shared environment and a person's location within a community. This makes individual consent alone insufficient; governance must acknowledge collective rights and control. Current data governance mechanisms, largely built around the European Union General Data Protection Regulation (GDPR) from 2016, the US Health Insurance Portability and Accountability Act (HIPAA) from 1996, and the US Genetic Information Nondiscrimination Act (GINA),^8^ however, are ill-suited to address the emerging challenges posed by continuous, and ideally passive exposure monitoring [8, 9].

Technological responses such as federated learning, homomorphic encryption, and blockchain-based consent tracking have been proposed to enhance data protection without impeding scientific collaboration [8, 28, 29]. However, such approaches require substantial infrastructure, transparent communication, and ethical governance frameworks to ensure they serve participants in addition to legitimate institutional interests.

### Informed consent and ongoing engagement

**Fig. 3:**
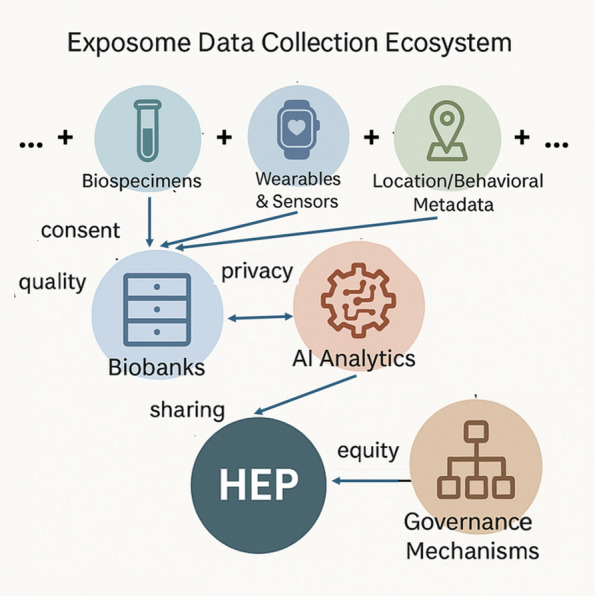
Exposome data collection ecosystem. A systems diagram showing connections between biospecimens, wearables, location tracking, biobanks, AI analytics, and governance mechanisms, each tagged with associated ethical challenges like consent, privacy, equity, and oversight

Informed consent is foundational to research ethics, yet its conventional model based on discrete decisions made at a single point in time is increasingly inadequate in the context of exposome studies. Exposome research entails repeated, long-term data collection and the potential for wide-ranging future data uses, often unanticipated at the time of initial consent (Fig. 3). This necessitates a shift toward broad, dynamic, or tiered consent models that offer greater flexibility and, at least for some approaches, more participant control over time [23, 30]. In line with Mascalzoni et al. [30], consent should be understood not merely as a one-time transaction or digital toggle, but as a dialogic and relational practice. Especially in longitudinal studies involving children, re-consent should function as a renewed ethical conversation, accommodating evolving identities and risks.

Dynamic consent systems, which leverage digital platforms to enable ongoing communication and re-consent, have gained traction in biobanking and are well-suited to the longitudinal and layered structure of exposome research. These models support participant autonomy, allow for preference updates, and facilitate re-contact in the case of incidental or actionable findings [21, 31]. A shift toward dynamic consent models is necessary because the longitudinal, discovery-driven nature of exposome research means that future data uses and potential findings are inherently uncertain at the outset. A one-time, static consent form cannot adequately cover decades of monitoring or the unforeseen implications of linking new data streams, requiring an ongoing relationship with participants. However, dynamic consent systems are not without limitations. They require substantial investment, raise concerns about digital exclusion, and can overburden participants if not carefully designed. Moreover, special populations, such as children enrolled prenatally or neonatally, raise distinct ethical questions about deferred autonomy, re-consent at adulthood, and parental representation [9].

Building on the insights of Safarlou et al. [21], exposomics consent processes should adopt a relational, dialogic model that treats participants as evolving agents with longitudinal rights over their data. Institutional frameworks (such as Institutional Review Boards, IRBs) should support re-consent procedures and digital interfaces that enable both re-engagement and selective opt-out, while addressing digital exclusion risks.

### Equity and environmental justice

Exposome research holds promise for addressing long-standing health disparities by illuminating the cumulative environmental burdens disproportionately borne by disadvantaged communities. However, unless explicitly designed with justice in mind, exposome research risks perpetuating extractive scientific practices in which vulnerable populations are monitored but not meaningfully engaged or empowered [25].

Participatory governance, such as Community Advisory Boards (CAB, see Operational Pillars: Ethics-by-Design for HEPs section) with real authority, is essential because exposome findings directly reflect socio-environmental conditions (e.g., pollution sources, housing quality) that are matters of collective, not just individual, experience and concern. Without shared control over the research process, the power to define problems and solutions remains with external actors, risking extractive outcomes.

Environmental justice concerns are particularly salient in communities historically exposed to industrial pollution, substandard housing, and occupational hazards. The “socio-exposome” framework emphasizes that structural inequality, rooted in race, class, and geography, shapes not only exposure but also susceptibility and health outcomes [26]. Exposome research must therefore incorporate community-engaged and/or participatory approaches, through community-informed formulation of study questions and engagement for community-relevant implications and solutions, and in some cases even full on community-based participatory research (CBPR). This will democratize knowledge production, empower affected communities, and promote community-defined benefit [32, 33].

Ethical engagement also requires consideration of collective rights, such as a community’s right to know, to govern how data is used, and to determine acceptable trade-offs between research participation and potential risks of stigmatization (e.g., labeling, exclusion and/or differential treatment of affected populations) or surveillance (e.g., monitoring or tracking—with re-identification feasibility—beyond original research purpose or without community governance).

Other ethical concerns include potential power asymmetries that may arise when those who control large exposomic datasets and the tools to analyze/use the data may wield significant influence over what insights are generated and whose risks are prioritized. Historically, environmental health research sometimes marginalized the very communities most affected by pollution or toxic exposures, leaving them with little say in research agendas or benefit from results [34].

Power asymmetries are central at the community level. The identification of an “exposure hot-spot” is not an ethical problem in itself; the problem arises from the power dynamics of how, why, and by whom this identification is made and used. If research is conducted on a community rather than with it, the findings risk being used in ways that reinforce existing injustices—for example, through stigmatization, economic disinvestment, or policy neglect—particularly when governmental or institutional actors fail to act on the evidence.

The primary ethical concern, therefore, is not merely the risk of marginalization but the systemic failure of institutions to translate evidence into protection and remediation. Ethical responsibility for change lies with public authorities, regulators, and funders, who must ensure that exposomic findings lead to preventive and corrective action. Communities cannot and should not bear that burden alone; rather, their role is to participate in shaping research priorities, interpreting results, and holding institutions accountable.

One aspect of power asymmetry is global (international) inequity. The envisioned Human Exposome Project will likely be driven by well-resourced nations, but environmental risks are global. Ethically, the global South must be a partner from the start, not just a beneficiary or an afterthought. While affluent nations may shoulder the costs of a Human Exposome Project, broad global collaboration and data-sharing under equitable terms are critical so that all populations benefit from the outcomes.

Global (as in international) equity thus must be embedded at the design stage, enabling the Human Exposome Project to avoid replicating the geographic and economic asymmetries that have constrained genomic data diversity. Similarly, as is discussed in the field of global genomics equity [35, 36], global equity in exposomics at the level of benefit sharing is also critical, for example, to ensure that not just high-income countries (HICs), but also low- and middle-income countries (LMICs) benefit from the outcomes of the HEP. In addition, there are a range of challenges associated with inequitable international research partnerships^9^ [37] (e.g., funding and resources inequalities, under-representation of LMIC in research programs, conferences and partnerships, issues with transparency in research motives and objectives, bilateral ethics review requirement), which also need to be considered in order for the global public–private partnership that is proposed to advance HEP, to be successful.

Finally, equity must also inform the interpretation and communication of results. Without explicit design to counteract structural bias, exposomic tools, particularly when driven by AI, risk reinforcing disparities in risk estimation and surveillance. Participatory governance and community co-design can mitigate these risks and transform data generation into instruments of environmental justice [38].

### Governance and oversight

The regulatory infrastructure for exposome research remains underdeveloped. Institutional Review Boards (IRBs) and ethics committees often lack the expertise to evaluate multi-omic, longitudinal, sensor-based research designs, particularly when data flows across institutions, borders, and time [8, 39]. Current frameworks struggle with challenges such as long-term consent validity, storage of data that could be re-identified, and the scope of ethical review when commercial partners or AI-based analytics are involved. An important next step is building institutional capacity for ethics oversight in exposome research. This includes dedicated training for IRBs on the unique ethical dimensions of exposomics such as longitudinal data sensitivity, the use of AI, and the (psycho)social implications of environmental data. We emphasize the importance of: ongoing training for researchers and IRB members, clear informed consent, including respecting the participants preference to whether or not receive certain types of (secondary) findings; providing access to counseling and psychosocial follow-up with participants when needed (i.e., to communicate identified risks similar as is done with genetics research [40, 41]).

**Table 1:** Comparative ethical challenges of the Human Genome and a Human Exposome Project

Ethical domain	Genome (HGP)	Exposome (HEP)
Data sensitivity	DNA sequences (immutable)	Multi-modal, behavioral + biological
Consent models	Static/broad consent	Dynamic/tiered/re-consent
Privacy risks	Genetic discrimination	Location inference, re-identification
Commercialization	Patenting genes, DTC kits	DTC exposure tracking, environmental AI
Equity concerns	Ancestry/population disparities	Environmental racism, exposure burdens

This table contrasts the ethical complexities of Human Genome Project (HGP)-based genomics with those emerging in the Human Exposome Project (HEP). It highlights how the exposome introduces greater data heterogeneity, dynamic consent requirements, higher privacy risks due to environmental traceability, and structural justice issues tied to ecological exposure disparities

Equitable governance should also be considered to overcome potential power asymmetries and environmental justice concerns as outlined in the previous section [42]. Research by Bhuller et al. [38] calls for explicitly integrating ethical principles such as proportionality, transparency, and precaution into environmental health decision-making. This aligns with the need for an exposome-specific governance model that addresses the long latency and distributive effects of environmental harms. The ethical governance criteria articulated by Bhuller et al. include procedural fairness, inclusiveness, and respect for pluralism. These principles require that decision-making processes be transparent, responsive to stakeholder concerns, and deliberative in handling value conflicts. Importantly, governance must also support adaptive capacity and accountability mechanisms to ensure public trust and ethical legitimacy over time. In practice, this means involving community representatives, ethicists, and diverse stakeholders when developing exposome AI models and when interpreting their outputs. It also means guarding against surveillance abuses: pervasive monitoring via wearables or sensors can quickly slip into privacy intrusion or even coercion if not bounded by strict ethical guidelines. Safeguarding autonomy and dignity is paramount when individuals are contributing personal exposure data.

Applied to exposome-based regulation, these criteria call for co-design with exposed communities, environmental-justice advocates, and other nontraditional data users so that problem formulation, data-collection priorities, and interpretation/communication of findings reflect lived realities; governance should institutionalize participation through stakeholder co-review, community data-use agreements, and transparent benefit-sharing, with iterative ethical impact assessments to anticipate and mitigate risks such as surveillance, stigmatization, and inequitable burdens.

Proposals have emerged for a dedicated Exposome Ethics Consortium, modeled after the ELSI program, to provide guidance, coordinate policy, and develop anticipatory governance strategies [9, 10]. Townsend et al. [43], while about pandemics, make a compelling, referenced case for the necessity and structure of new international ethics bodies to address complex, large-scale health challenges. Such a body could support the development of best practices, assist in resolving conflicts over secondary use, and promote international alignment through ethical standard-setting initiatives akin to the FAIR principles^10^ and the Global Alliance for Genomics and Health.

**Table 2:** Major exposome research programs and ethical features

Program	Region	Focus areas	Ethical components included
HELIX	EU	Child exposome, cohorts	GDPR, community input (limited)
EXPOsOMICS	EU	Air/water pollution	Data protection frameworks
EXIMIOUS	EU	Immune-mediated diseases	Informed consent, FAIR data compliance
HHEAR	US	Environmental biomonitoring	Consent, return of results discussion
EHEN	EU	Network of 9 projects	Ethics and Law working group, harmonization efforts

This table summarizes key international exposome initiatives, their geographic scope, scientific focus, and notable ethical components. The table illustrates how European and U.S. programs have progressively integrated data governance frameworks, community engagement, and ethical harmonization efforts—laying the groundwork for a coordinated ethical infrastructure

### Actionability and the return of results

A distinctive ethical challenge in exposome research is the question of actionability when and how to return findings to participants. Many exposome signals are of uncertain clinical relevance, context-dependent, and lacking in clear exposure thresholds. This makes it difficult to determine when disclosure is ethically warranted or beneficial. Moreover, sharing of findings that suggest increased risk (e.g., elevated levels of endocrine disruptors or air pollution exposure) may provoke anxiety or stigma without offering clear pathways for remediation [10, 44].

The concept of actionability must be expanded for the exposome because the ability to act on a finding (e.g., 'high lead exposure') is intensely context-dependent. For an individual with resources, action may mean installing a water filter. For a low-income community, the only meaningful action is collective political advocacy for pipe replacement, a distinction that individual-centric return-of-results frameworks often miss.

An example of actionability is when and how to return findings to participants, and what constitutes an "*actionable*" insight. Exposome signals are often of uncertain clinical relevance, context-dependent, and lack clear thresholds. To navigate this complexity, it is essential to distinguish between different types of actionability, each with distinct ethical implications. As outlined in Table 3, we categorize actionability into four overlapping types: personal, clinical, community-level, and policy-level. This typology is presented as a proposed analytical framework to structure discussions of actionability within exposome research. It has been developed through conceptual synthesis rather than participatory validation and should therefore be viewed as an initial scaffold to be refined through future collaboration with communities, policymakers, and other stakeholders.

**Table 3:** A typology of actionability for exposome research findings

Actionability type	Definition and context	Example findings	Key ethical implications and considerations
Personal actionability	Findings where an individual can take direct action to reduce exposure or risk, often dependent on personal resources and agency	- Elevated levels of a dietary chemical (e.g., BPA) - Personalized air pollution exposure peaks linked to a specific commute	- *Risk of "individualizing blame"*: Must avoid framing structural problems as individual responsibilities. Communication should emphasize systemic sources of exposure - *Resource dependency*: Actions (e.g., buying filters, changing diet) may not be feasible for all. Must provide context and avoid assumptions
Clinical actionability	Findings that may inform clinical monitoring, early detection, or preventive healthcare strategies	- Biomarker profile suggesting increased risk for asthma exacerbation - Exposure signature linked to accelerated cognitive decline	- *Uncertain clinical utility*: Many exposome signals are probabilistic and lack established clinical thresholds. Requires clear communication of uncertainty to avoid overdiagnosis or anxiety - *Integration into care*: Needs pathways for clinical validation and collaboration with healthcare providers to be meaningful
Community-level actionability	Findings that empower a community for collective advocacy, local intervention, or resource mobilization	- Identification of a local "hot-spot" for lead in drinking water - Data showing disproportionate airborne toxicant exposure in a specific neighborhood	- *Empowerment vs. stigmatization*: The primary ethical goal is empowerment. Findings must be co-interpreted and controlled by the community to prevent labeling and economic harm - *Community governance essential*: Requires strong participatory governance (e.g., CABs) to ensure data is used as a tool for justice, not further marginalization
Policy-level actionability	Findings that provide evidence for regulatory changes, public health guidelines, or environmental remediation at municipal, national, or international levels	- Population-level data linking a specific industrial chemical to adverse birth outcomes - Evidence that current air quality standards are insufficient to protect vulnerable groups	- *From evidence to action*: Requires transparent Evidence-to-Decision (EtD) frameworks that weigh benefits, harms, and equity - *Precautionary principle*: Should guide action in the face of uncertain but plausible risk, especially for widespread exposures - *Justice imperative*: Policy responses must prioritize reducing disparities and protecting the most vulnerable

This table categorizes potential actionable insights from exposome data, providing examples and key ethical implications to guide researchers and stakeholders in the responsible return of results. The typology emphasizes that actionability is context-dependent and must be evaluated through an ethical lens to avoid harm and promote justice. *Note* This typology is adapted from a contextual framework for actionability in exposome research [14].

Recent scholarship emphasizes the need for a broader concept of actionability, one that includes psychosocial and contextual dimensions [21]. This includes distinguishing between personal actionability (e.g., changing diet or housing) and collective actionability (e.g., community mobilization or policy change), and recognizing that what is actionable for one population may not be for another due to structural constraints. Robust ethical frameworks are needed to guide the return of results in a manner that respects autonomy, avoids harm, and supports health-promoting behavior without shifting responsibility to individuals for problems rooted in structural injustice.

## Artificial intelligence and exposome ethics

The integration of artificial intelligence (AI) and machine learning into exposome research has dramatically expanded the ability to model complex exposure–health relationships. These technologies enable the analysis of high-dimensional, multimodal data, including environmental, molecular, behavioral, and sociodemographic variables, to support predictive risk modeling and personalized prevention [45, 46]. However, their use raises pressing concerns regarding algorithmic fairness, transparency, and accountability. In the following sections, we dive deeper into the ethical challenges around the use of AI in Exposome research, addressing, e.g., risk of biases, transparency and ethical accountability, and providing recommendations for mitigating AI-related ethical risks. This is a major difference to the HGP—the HGP did not need AI, the HEP is not possible without. However, the HEP therefore also comes with ethical burden of AI. We stress the “*ethics-by-design*” principle [47] for AI as formulated by UNESCO's 2022: Recommendation on the Ethics of Artificial Intelligence (UNESCO [9826] Document code: SHS/BIO/PI/2021/1),^11^ which embeds human rights, equity, and environmental sustainability across the full AI lifecycle. In practice, this means participatory co-design, documented impact/risk assessment and due diligence, transparency/traceability and auditability, human oversight with accessible redress, and continuous post-deployment monitoring to ensure accountability.

While general principles of AI ethics, i.e., fairness, transparency, accountability, are well established, their implications in exposome research are distinct. The integration of high-dimensional biological, environmental, and social data makes issues such as multimodal bias, re-identification, and interpretability not only technical but deeply ethical, as they directly affect environmental-justice outcomes.

Bias can enter exposome-AI pipelines at multiple stages: during data collection (e.g., under- or overrepresentation of potentially vulnerable groups), preprocessing (e.g., imputation assumptions), or modeling (e.g., overfitting to dominant exposure profiles). These biases can perpetuate structural inequities in health outcomes by misrepresenting risks for historically marginalized populations or reinforcing flawed assumptions about causality [8, 48]. Notably, training models predominantly on urban, European, or affluent cohort data may yield tools that are ill-suited or even harmful when applied to rural, low-income, or racially diverse populations [26].

Moreover, the “*black box*” nature of many AI models challenges both scientific transparency and ethical accountability. While explainable AI (xAI) techniques [49] such as SHAP (SHapley Additive exPlanations) are beginning to demystify model decisions, there is a long way to go before the rationale behind exposome-based predictions is both scientifically robust and ethically communicable [21, 50]. To mitigate these risks, exposome research should adopt algorithmic audit protocols, prioritize inclusivity in training data, and build interdisciplinary teams that include ethicists, social scientists, and community representatives during model development and deployment. Thus, the ethical governance of AI in exposomics must be evaluated not in isolation but as part of the broader exposome-ethics architecture connecting algorithmic accountability to dynamic consent, data sovereignty, and Evidence-to-Decision frameworks.

### AI’s expanding role in exposome research

Recent advances in AI have positioned it at the center of exposome science [51]. Exposome research produces high-dimensional, heterogeneous datasets far beyond the scale of traditional epidemiology, including sensor readings, multi-omics profiles, geospatial data, and clinical records [52]. Mapping this “*labyrinth of data*” was once deemed practically impossible, but AI is now “*changing everything*” by enabling deep learning models to harmonize disparate data types, extract hidden patterns from millions of features, and predict the health impacts of complex exposure mixtures [53, 54]. In essence, AI may provide the computational power and algorithms needed to recognize patterns in the noise—detecting associations between lifelong exposure profiles and disease risks that humans or simpler statistical methods might miss.

One of AI’s most transformative capabilities is integrating multimodal exposomic data into unified analyses. Sophisticated machine learning architectures (sometimes dubbed “*exposome intelligence*”) [51] can weave together inputs as varied as mass spectrometry chemical signatures, genomic and epigenomic data, personal sensor readings, satellite-derived pollution metrics, and even social media or socioeconomic indicators [55]. By fusing these data, AI systems construct a more holistic portrait of an individual’s exposome than any single data type could provide. For example, neural networks can concurrently analyze a person’s metabolomic biomarkers alongside their neighborhood air quality and lifestyle factors, uncovering complex interactions between exposures and biology. Such data fusion is critical for exposomics, which seeks to understand how multiple exposures combine to influence health. Indeed, regulators note that a key development in AI is the ability to handle multiple modalities in complex, high-dimensional datasets, offering more realistic risk assessments that consider the full spectrum of data types (omics, genetics, environmental measures, etc.) rather than siloed factors.

AI could also drive predictive modeling in exposome research. Machine learning models excel at finding nonlinear relationships and high-order interactions, enabling the prediction of health outcomes based on exposure profiles. Deep learning methods, for instance, can analyze complex mixtures of environmental chemicals to predict toxicity or disease risk, moving beyond the simplistic one-exposure–one-outcome paradigm of traditional toxicology. By training on large-scale longitudinal datasets, AI algorithms can begin to infer causal links from correlations, especially when combined with techniques for causal inference on long-term exposure and health data. This opens the door to identifying which patterns of early-life exposures are truly driving later disease, or which combinations of lifestyle and chemical factors synergize to elevate risk. Moreover, AI models can be personalized by incorporating individual attributes (genetics, age, sex, behaviors), yielding individualized risk scores and exposure recommendations. For example, an AI could analyze a person’s unique exposome and predict their likelihood of developing asthma or cancer, then suggest tailored preventive actions (such as avoiding certain pollutants or dietary changes). In short, AI provides the toolkit to transform the exposome concept into actionable risk prediction, mechanistic insight, and prevention strategies. Pairing the exposome with AI’s integrative and predictive capacity offers a path to proactively safeguard health akin to HEP that could transcend the HGP in impact, especially since the exposures are in many ways addressable.

However, with these powerful capabilities come new ethical responsibilities. The very qualities that make AI invaluable, its hunger for data, pattern-hunting prowess, and complexity, also introduce ethical challenges that exposome researchers must confront. Next, we examine these challenges, including algorithmic bias, transparency and explainability, data provenance, and power asymmetries. Addressing these issues is essential if AI is to fulfill its promise in exposomics responsibly and with public trust.

### Ethical considerations for AI in exposomics

AI is the indispensable 'guidance computer' for the ambitious HEP, enabling the data fusion, predictive modeling, and real-time surveillance needed to move from dream to reality [53]. However, this very power means that AI does not merely introduce generic ethical concerns into exposome research; it intensifies and qualitatively alters the specific ethical dimensions outlined in "Key ethical dimensions of exposome research" section. This section moves beyond a general overview of AI ethics to provide a critical analysis of how AI's key capabilities for the HEP interact with its foundational ethical challenges.

#### Algorithmic bias and data representativeness

Algorithmic bias in AI refers to systematic errors or skewed outcomes that disproportionately affect certain groups. In exposomics, bias can creep in at multiple stages of the AI pipeline. During data collection, if certain populations (for example, low-income or minority communities) are underrepresented in exposome studies, AI models may be trained predominantly on data from more privileged or convenient cohorts. The opposite is true when studies over-represent certain populations. Misrepresentation can thus lead to models that perform well for well-characterized groups but poorly for others, effectively neglecting encoding existing health disparities into the algorithms. These biases can perpetuate structural inequities in health outcomes by misrepresenting risks for historically marginalized populations or reinforcing flawed assumptions about causality [8, 48]. As Safarlou et al. [8] note, exposome research often relies on big-data approaches, but if those data reflect biased sampling or societal inequalities, the conclusions will also be biased. A model heavily trained on urban European cohorts, for instance, may misjudge risks for rural or non-European populations; such a tool could be “*ill-suited or even harmful*” when applied to communities with different exposure profiles. This is not just a statistical concern but a normative one: it raises issues of justice and fairness. Marginalized groups—who often face higher pollution and exposure burdens—could be further disadvantaged if AI models undervalue or mischaracterize the risks they face.

Rigorous protocols for algorithmic fairness are non-negotiable because AI models in exposomics are trained on multimodal data (environmental, behavioral, biological) that often reflects and can amplify existing societal biases. For example, if pollution sensors are disproportionately deployed in certain neighborhoods, the resulting models will generate skewed exposure assessments for entire populations, perpetuating environmental injustice.

It is important to distinguish technical bias [56] from ethical bias in this context. AI scientists use “*bias*” in the technical sense (systematic error or deviation from truth in a model’s predictions). But in ethics, bias refers to unjust or prejudicial treatment of people. These two meanings intersect in exposome AI. A poorly calibrated model might be technically biased (e.g., overestimating everyone’s exposure to a toxin), but more worrying is if errors affect groups unequally—say, under-predicting dangers in a minority community due to sparse data, which would be a normative bias resulting in unfair outcomes. Ensuring data representativeness—that training data cover diverse populations and contexts—is therefore an ethical imperative. Otherwise, AI could reinforce structural inequities in environmental health, making invisible the exposures of already underserved communities. The principle of fairness demands that AI models neither exacerbate existing disparities nor create new ones.

#### Holistic data fusion vs. privacy and data sovereignty

AI's need to fuse highly personal data (GPS, biospecimens, social data) creates an unparalleled privacy challenge. The exposome's "inherently communal" data (e.g., neighborhood air quality) blurs lines between individual and community sovereignty. AI makes re-identification from anonymized datasets not just a risk, but a near certainty with implications not only for the individual, but the entire community, demanding new models of collective data governance.

#### Transparency and explainability

The complexity of AI models (especially deep neural networks) often makes them “*black boxes*”—their inner workings are not transparent even to the scientists who deploy them. This opacity poses a serious ethical and scientific issue in exposomics: stakeholders may rightly ask, “*Why should we trust an AI’s prediction about exposure X causing disease Y?*” If the model cannot provide an explanation, it undermines scientific transparency and accountability. In regulated domains like environmental health, decisions must be explainable to be credible; otherwise, we risk basing public health policies on inscrutable algorithms. Lack of explainability also frustrates participants’ rights: individuals subjected to AI-driven exposure assessments or recommendations have a right to know the reasoning behind decisions that affect their lives.

There is growing work on explainable AI (xAI) techniques in health, such as SHAP (SHapley Additive exPlanations) values or interpretable model architectures, to illuminate how models reach their outputs [29]. Early efforts show promise in demystifying AI decisions—for example, highlighting which exposure variables most influenced a prediction—but as we and Safarlou et al. observe, there is still “*a long way to go*” before exposome-based AI predictions are both scientifically robust and ethically communicable to non-experts [21, 50]. The danger is that without adequate explainability, AI could erode trust: communities might suspect the models are “*magic*” or, worse, biased against them, and experts cannot easily validate the findings. Transparency must therefore be a design goal: from open algorithms and documentation to communication strategies that translate complex model results into understandable insights. This links directly to the principle of respect for persons—treating participants not merely as data sources but as partners who deserve to understand how and why an AI might flag their neighborhood’s water as high-risk or their personal exposure profile as concerning.

#### Data provenance and quality

AI’s strength in exposomics—finding patterns across massive datasets—is also a potential weakness if those data are of dubious quality or origin. Data provenance refers to knowing the source and history of data: how were exposure data collected? By whom? Using validated instruments or protocols? How were the data cleaned and merged? These questions are crucial because an AI model is only as reliable as the data feeding it. If data sources include unverified community reports, experimental -omics assays with batch effects, or sensor readings with calibration drift, the model may learn spurious patterns or be misled by noise. Ethical exposome science demands careful data curation and validation before AI modeling, to avoid drawing false conclusions that could misguide health interventions.

Closely related is the issue of data quality control. In traditional epidemiology, researchers painstakingly design studies and verify data, but in big exposome datasets (often repurposed from many studies or devices), ensuring consistency and accuracy is challenging. AI can inadvertently mask data quality issues because complex models might fit artifacts that humans overlook. Transparent reporting of data provenance (what data were used, with what uncertainties) is therefore essential for both ethics and reproducibility. Emerging frameworks in evidence-based toxicology emphasize rigorous validation of new methods, including AI models, by checking them against reference datasets and known benchmarks. Applying such validation frameworks to exposome AI means building in checks for data integrity and model performance before claims are made. Ethically, this aligns with the principle of veracity in science—an obligation to ensure that results are truthful and not products of unchecked garbage-in, garbage-out dynamics, wasting resources and burdening participants without purpose. It also enables auditability, since independent reviewers (or communities) can trace how an exposure conclusion was reached from the raw data upwards.

#### Power asymmetries and justice

The use of AI in exposomics also brings questions of power and equity to the forefront. Data is power: as indicated above, entities that control large exposomic datasets and AI tools—often well-funded research institutions, tech companies, or government agencies—may exert substantial influence over what insights are produced and whose risks receive priority. This raises concerns about power asymmetries between data generators (e.g., participants or communities being monitored) and data analysts/owners^12^ [57, 58]. There is a moral risk that AI could exacerbate this if, for instance, AI-powered surveillance technologies heavily monitor certain neighborhoods without community governance, or if AI-derived insights primarily benefit private companies or wealthy populations.

AI could drive global inequity, for example, when AI models are developed solely in high-income settings [59], they might not address exposure concerns prevalent in low-income countries (for example, indoor air pollution from cookstoves or unregulated chemical dumping). AI models could also highlight certain communities as high-risk, leading to stigma, unwanted surveillance, or economic disadvantages.

#### Causal inference and personalized risk vs. justice and equity

The growing use of wearable sensors—personal devices that continuously capture physiological or location-linked exposure data—together with ambient environmental sensing networks (e.g., air-quality monitors, satellite feeds, public sensor grids) creates a new governance frontier. While many environmental sensors collect only public or non-identifiable data and therefore fall outside traditional human-subjects regulation, ethical complexity emerges when exposome research links these datasets to personal biospecimens, geolocation traces, or behavioral profiles. Such integration blurs the line between population-level monitoring and individualized surveillance.

The capability to fuse personal and ambient data can empower communities with real-time evidence for advocacy and exposure mitigation, yet it also opens the door to ambient surveillance—continuous, often opaque monitoring by corporations, insurers, or state agencies that use exposomic insights to profile populations or infer risk without consent. The ethical question, therefore, is not whether environmental sensing occurs, but who controls the infrastructures that collect, integrate, and interpret such data, and who benefits from their insights. Addressing this requires governance mechanisms that ensure transparency, community participation, and public accountability for both the use and reuse of sensor-derived exposomic information.

#### Real-time surveillance vs. governance and power

The vision of wearable sensors and real-time hazard surveillance, powered by AI, creates a profound governance gap. This capability could empower communities with data for advocacy, but it also opens the door to ambient surveillance by corporations or governments. The ethical question becomes: who controls the surveillance infrastructure, and who benefits from its insights? This exceeds the governance challenges of traditional biobanking.

#### Actionability and the "*black box*" vs. trust and participation

The “*black box*” challenge of AI, where model outputs cannot be readily explained, poses a specific barrier to actionability in exposome research. When risk predictions or recommendations lack interpretability, participants and policymakers cannot meaningfully act on them, undermining both ethical transparency and public trust. Explainable AI (xAI) approaches are therefore essential not merely for technical validation but for enabling informed decision-making by communities and regulators.

According to the principles proposed by Bhuller et al. [38] as outlined in the governance section above, justice in AI-enabled exposomics requires inclusive governance over both data collection as well as model development and interpretation, ensuring that affected communities and diverse stakeholders have a meaningful role. Safeguards must prevent AI-driven surveillance from crossing into privacy violations or coercion, particularly when continuous monitoring via wearables or sensors is involved. Protecting autonomy and dignity is essential when personal exposure data inform AI models. In summary, ensuring justice in AI-enabled exposomics requires actively redressing power imbalances by inclusion, shared decision power, and vigilant oversight to avoid “*exposome marginalization*,” and so that AI tools serve the many and not just the powerful few.

In response, we advocate for ethics-by-design AI development, aligned with principles of proportionality, inclusiveness, and explainability. Algorithmic audit protocols, transparency standards, and participatory model evaluation must be built into exposome AI pipelines to uphold scientific integrity and social trust.

### Building trust through ethical AI governance

For AI to truly succeed in exposome research, the public and scientific stakeholders must trust these tools and their outputs. Building trust is both an ethical obligation and a practical necessity: without trust, study participants may hesitate to share data, regulators may reject AI-derived evidence, and communities may resist public health interventions driven by AI findings. As Hartung et al. [60] observed in a regulatory science context, “*trust is key in AI*”, but defining and earning that trust requires deliberate effort. The following approaches are recommended to foster trustworthy AI-enabled exposome science, echoing emerging frameworks in AI validation, evidence-based toxicology, and participatory governance:

#### Participatory model development

Involve communities and stakeholders from the outset when designing AI models and exposome studies. When people see their lived experience and values reflected in the way an AI model is built and used, they are more likely to trust its findings. Participatory design also helps to surface ethical concerns early (e.g., cultural sensitivities about data collection or potential misuse of predictions) and ensures the research addresses real community needs, not just academic curiosity.

#### Interdisciplinary ethics oversight

AI-enabled exposomics should operate under interdisciplinary ethical oversight (as described above) throughout the project lifecycle. Notably, Institutional Review Boards and regulatory committees are still catching up to the complexities of AI in longitudinal environmental health studies; having specialized ethics working groups can fill this gap and provide guidance tailored to exposomics and AI, to ensure that, as the AI models evolve, they remain aligned with social values and rights.

#### Transparency standards and auditability

To bolster trust, exposome AI models should adhere to high transparency standards. This includes publishing model methodologies, performance metrics, and known limitations openly. Complex AI systems should, where possible, provide explainable outputs or user-friendly summaries so that researchers and participants can understand what factors drive the predictions. In addition, adopting algorithmic audit protocols is crucial. Independent auditing of AI models, i.e., checking for biases, errors, and appropriate use of data, can validate that the tools perform as advertised and identify unintended harmful behavior. For example, audits might reveal if an AI under-predicts risk for a certain minority group or if it relies on an outdated proxy (such as using ZIP code as a surrogate for socioeconomic status in a way that could discriminate). By instituting routine audits and publishing the results, researchers demonstrate a willingness to be held accountable. In the spirit of evidence-based validation, such audits and validations should be continuous, not one-off. Hartung and colleagues propose “*e-validation*” frameworks [61, 62] where AI itself aids in continuously validating new approach methods in toxicology—similarly, exposome AI could employ continuous monitoring agents that flag when a model’s predictions start drifting or when new data fall outside the training distribution. In summary, transparency and auditability turn the black box into a glass box, enabling oversight bodies and the public to look inside and be confident that the AI is robust and fair [63].

#### Inclusivity and fairness in governance

As described above, inclusive governance structures are needed to address power asymmetries and ensure fair benefit-sharing. Governance here means both the policies that guide exposome AI use and the bodies that make decisions. Inclusive governance builds trust by showing that exposome AI is being guided by ethical principles and by people from all walks of life, not just a technocratic elite.

#### Validation and regulatory alignment

To gain trust at the institutional level, AI methods in exposomics must earn regulatory acceptance as credible evidence. Regulatory science is increasingly grappling with how to evaluate AI models for use in risk assessment and public health decisions [60]. Ensuring that exposome AI models meet rigorous validation criteria (sensitivity, specificity, reproducibility, etc.) will make regulators and policymakers more comfortable relying on them. Researchers should collaborate with regulators early to identify what evidence is needed for an AI model to inform, say, chemical safety standards or environmental exposure limits. This might involve benchmarks and cross-validation against existing gold-standard methods, registering models and their intended use (model documentation akin to a pre-registered protocol), and even certifying algorithms similarly to medical devices. The FDA and other agencies have begun outlining principles for AI in healthcare, emphasizing issues like reliability, explainability, and bias mitigation—these should be heeded in exposome applications as well. By aligning AI development with evidence-based toxicology practices (which stress systematic evidence review and “*weight of evidence*” approaches), scientists can demonstrate that their models are not black magic but grounded, tested tools. Over time, successful case studies of AI improving exposure assessment or predicting health outcomes will build a track record that fosters trust among skeptics. As one global summit concluded, ongoing dialogue between AI developers and regulators, along with adaptable oversight frameworks, is key to successful AI adoption in any high-stakes domain.

#### Education and empowerment

Finally, building trust in AI-driven exposomics is aided by educating both the public and the scientific community. Participants in exposome studies should be offered clear information about what AI is doing with their data and what the results mean for them. This could include community workshops on interpreting exposure scores or understanding probabilities, thereby improving health literacy around the exposome. On the scientific side, training the next generation of environmental health researchers in AI techniques and ethics is crucial. Equipping experts who can straddle data science and ethical governance will ensure that future innovations remain human-centric. Hartung [64] emphasizes training environmental health scientists in data ethics, causal modeling, and AI methods so that complex model outputs can be translated into actionable strategies for prevention. Such interdisciplinary expertise itself builds trust, as teams can internally evaluate and communicate AI results responsibly. Moreover, when communities see researchers taking ethics seriously—for example, being transparent about uncertainties or involving ethicists at town hall meetings—it engenders confidence that “*these AI tools are on our side*.”

In conclusion, AI holds immense promise for advancing exposome research by managing complexity and unveiling new insights into how our environments shape health. Yet, realizing this promise requires more than technical prowess; it demands an ethical framework that addresses bias, ensures transparency, respects all stakeholders, and builds a governance system worthy of public trust. Encouragingly, recent literature is converging on these needs: Safarlou et al. [10] urge rethinking exposome science toward actionable benefit for communities, Hartung [51] outlines how AI can enable a proactive HEP undergirded by robust validation and cross-sector cooperation, and Bhuller et al. [38] articulate ethical principles to guide risk decisions in the AI era. By weaving these insights into a coherent ethical approach, we can ensure that artificial intelligence in exposomics develops not only as a powerful scientific tool but as a trustworthy ally in the pursuit of health equity and environmental justice. The ultimate measure of success will be an AI-empowered exposome science that delivers actionable knowledge while upholding the values of transparency, fairness, and inclusivity, truly benefiting all in society.

As exposomics continues to integrate AI and multi-omics technologies [65], it presents an opportunity to connect more deeply with the broader omics landscape—particularly genomics. The ethical and analytical challenges explored in this section, from algorithmic fairness to data sovereignty, resonate strongly with issues already encountered in genomic research. Positioning exposome ethics within this broader translational omics framework reinforces the value of cross-disciplinary learning and policy harmonization. Moreover, it offers a timely contribution to efforts in human genomics to ensure that AI-driven, data-intensive science evolves with ethical foresight, social responsiveness, and a commitment to health equity.

## Emerging ethical frontiers in exposome research

As the Human Exposome Project advances technologically and methodologically, it faces a new generation of ethical challenges that stretch beyond traditional bioethics frameworks. These challenges are intensified by the convergence of exposomics with artificial intelligence, wearable technologies, and commercialization, and they raise fundamental questions about surveillance, fairness, and the role of profit in public health science. Beyond AI, exposome ethics must also anticipate ethical concerns emerging from sensor technologies, commercialization pressures, and surveillance risks. These challenges require coordinated ethical infrastructures. As discussed for AI Artificial Intelligence and algorithmic bias in the preceding chapter, addressing these emerging frontiers requires adaptive ethical strategies that keep pace with innovation and maintain a focus on justice, transparency, and public trust.

### Wearables, passive sensors, and the ethics of ambient surveillance

Exposome science increasingly relies on wearable and passive sensor technologies—such as personal air monitors, GPS trackers, biometric wearables, and smartphone apps—to capture high-resolution data on environmental and behavioral exposures [66]. While these tools offer unprecedented granularity, they also blur the line between research and surveillance.

Unlike traditional data collection methods, passive sensors may operate continuously and unobtrusively, potentially collecting information without the participant’s active awareness or ongoing consent. This raises ethical concerns about autonomy, dignity, and behavioral manipulation. Participants may change their behavior (consciously or subconsciously) when under observation—a phenomenon known as the Hawthorne effect—which can affect data quality and psychological well-being [8, 67].

Moreover, passive environmental data capture often includes sensitive contextual information—such as location, social interactions, or emotional states—that extends beyond the health domain and may pose risks of profiling, discrimination, or stigmatization. For example, repeated exposure measurements may reveal socioeconomic status or identify individuals residing in polluted or unsafe neighborhoods, raising the risk of unintended labeling or exclusion [9, 44].

These issues call for a rethinking of consent processes and data minimization principles. Researchers must balance scientific utility with participant dignity, ensure ongoing opt-in mechanisms, and establish clear boundaries for secondary use of sensor-derived data.

### Commercialization and the risk of data commodification

As exposome research becomes more technologically intensive, it increasingly intersects with the interests of commercial actors, e.g., sensor manufacturers, data analytics firms, biotechnology companies, and digital health platforms. These partnerships can accelerate innovation and expand infrastructure, but they also raise concerns about data ownership, profit-sharing, and the erosion of public trust [22, 68].

Unlike genomics, which has faced public controversies over patenting of gene sequences and the commercialization of ancestry and health tests, the exposome’s vulnerability to commodification has so far received less attention. Yet the risks are significant. Commercial interests may push for the development of direct-to-consumer exposome profiling kits or app-based exposure tracking services that promise personalized health insights without adequate clinical validation or ethical oversight [8, 9].

Furthermore, as exposome datasets gain value for insurance risk modeling, pharmaceutical development, or urban planning, they may be subject to opaque licensing agreements or proprietary analytics, distancing the research outputs from public benefit. This raises concerns about equitable access to innovation and the potential misuse of data for discriminatory purposes [69].

To safeguard the public ethos of exposome science, ethical governance must enforce transparency in commercial collaborations, ensure benefit-sharing with participants and communities, and prohibit exploitative data practices. Policy tools may include use limitations, compulsory data stewardship agreements, and ethics review standards tailored to public—private research consortia.

## A framework for ethical human exposome projects

The preceding sections have highlighted the unprecedented ethical challenges that arise from the scale, complexity, and social embeddedness of exposome research. These challenges demand more than compliance with traditional bioethics norms—they require the design of new frameworks for anticipatory, participatory, and justice-oriented research governance. In this section, we propose a conceptual and operational foundation for ethical HEPs, grounded in six guiding principles and three structural pillars that together aim to embed ethics throughout the research lifecycle.

### Guiding principles for ethical exposome science

#### Inclusivity

HEPs must be inclusive not only in cohort composition but in governance. This includes the equitable representation of historically marginalized groups in study design, data interpretation, and benefit sharing. Without such inclusivity, exposome research risks reinforcing existing health disparities under the guise of objective science [26].

#### Transparency

All elements of the HEP, from data collection methods and consent models to AI decision-making and commercial partnerships, must be disclosed in clear, accessible language. Transparency is not only a condition for informed consent but also a precondition for public trust [70].

#### Justice

Justice must operate at both procedural and distributive levels. Procedurally, communities must be engaged as co-designers rather than subjects. Distributively, the benefits of exposome research, including data insights, policy interventions, and technologies, must be shared equitably across participant groups [25, 32].

#### Reciprocity

HEPs must view participants not merely as data sources but as partners. Reciprocity entails offering timely, meaningful information in return, facilitating community health literacy, and committing to long-term engagement, especially when exposome research uncovers risks with no immediate remediation pathway [21, 44].

#### Precaution

Where scientific uncertainty exists, especially around low-level but persistent environmental exposures, HEPs should favor precautionary approaches that minimize harm. This includes reporting uncertain but potentially harmful exposures in ways that empower individuals and communities to act [8, 69].

#### Actionability

Ethics must not end at risk identification. HEPs should prioritize actionable knowledge, whether individual (e.g., exposure reduction), clinical (e.g., early diagnosis), or policy-level (e.g., environmental regulation). Moreover, actionability should be assessed through a contextual lens: what is actionable for a high-resource individual may not be for someone facing structural disadvantage [21].

#### Scientific integrity and methodological ethics

Ethical exposomics requires norms for data quality, reproducibility, and evidentiary sufficiency. Drawing on the principles of validation ethics [64], exposome science should employ structured, probabilistic validation processes and transparent evidence synthesis.

### Operational pillars: ethics-by-design for HEPs

To translate these principles into practice, we propose three institutional mechanisms:

#### Participatory governance structures

Establishing Community Advisory Boards (CABs) is essential to integrate lay perspectives, particularly from overburdened communities. These bodies should have real authority in decision-making, including study priorities, data use terms, and communication strategies. Successful models from environmental justice and CBPR research demonstrate that such structures are feasible and impactful [23, 25]. Establishing CABs is essential, but they must be granted real decision-making power, not serve a tokenistic function. This includes:

- Co-Design of Research Questions: CABs should be involved from the inception to ensure the research addresses community-prioritized health concerns, not just academic interests.
- Data Use Agreements and Veto Power: Communities, via their CABs, must co-create data governance agreements that specify allowable uses of data. This should include the right to veto certain secondary data uses (e.g., for commercial purposes or by entities perceived as hostile to community interests) that do not align with community-benefit principles.
- Interpretation and Communication Partnership: Communities should be partners in interpreting findings and developing communication strategies to ensure results are presented in a context that supports advocacy and avoids reinforcing disadvantage. This does not imply a veto over publication; rather, it establishes a process of collaborative review and contextual co-framing, whereby community representatives can provide input on wording, implications, and dissemination plans before submission or media release. The ultimate responsibility for publication integrity and scientific accuracy remains with the investigators, but the ethical goal is to prevent misinterpretation and foster mutual trust through shared communication planning.

#### Parallel ethics research tracks

HEPs should embed ethics research as a parallel track from the outset—akin to embedded social science in clinical trials or implementation research. These tracks can perform normative analysis, monitor participant perspectives, and audit the implementation of consent, privacy, and governance mechanisms [8]. This approach has been used in genomics and nanotechnology and is gaining traction in exposome-funded consortia.

#### Ethics oversight bodies with domain expertise

Traditional IRBs may lack the interdisciplinary expertise required to evaluate exposome protocols. We recommend the formation of Exposome Ethics Review Panels—either local or national—comprising experts in environmental health, data science, social science, ethics, and community engagement. These panels can issue ethical certifications, monitor compliance, and serve as public interlocutors.

To translate these principles into practice, we propose the establishment of an international Exposome Ethics Consortium (EEC). This body would function as a central coordinating hub, distinct from and complementary to local IRBs. Its primary role would not be to replace project-specific ethical review but to build capacity, set standards, and provide guidance for the unique challenges of exposome science that traditional IRBs are currently unequipped to handle.

The Consortium's structure and functions should include an international, multi-stakeholder governance board comprising ethicists, environmental health scientists, data governance experts, community representatives from environmental justice organizations, legal scholars, and policymakers. This ensures diverse perspectives are embedded in its guidance. Sustained funding must be a priority for national and international research agencies (e.g., NIH, European Commission) that sponsor large-scale exposome initiatives, recognizing that ethical infrastructure is as critical as technological infrastructure.

Key Functions should be:

- Developing Guidelines: Creating harmonized, international guidelines for dynamic consent, data sovereignty agreements, community benefit-sharing, and the ethical use of AI in exposomics.
- Training and Capacity Building: Providing specialized training modules and certification for IRB members and researchers on exposome-specific ethics, thereby supplementing and enhancing local oversight.
- Policy Advocacy: Acting as a unified voice to advocate for regulatory updates that accommodate longitudinal, sensor-based, and community-engaged research designs.
- Knowledge Repository: Maintaining a public repository of case studies, best practices, and model consent forms to support the global research community.

### Toward an ethical infrastructure for the exposome era

**Fig. 4:**
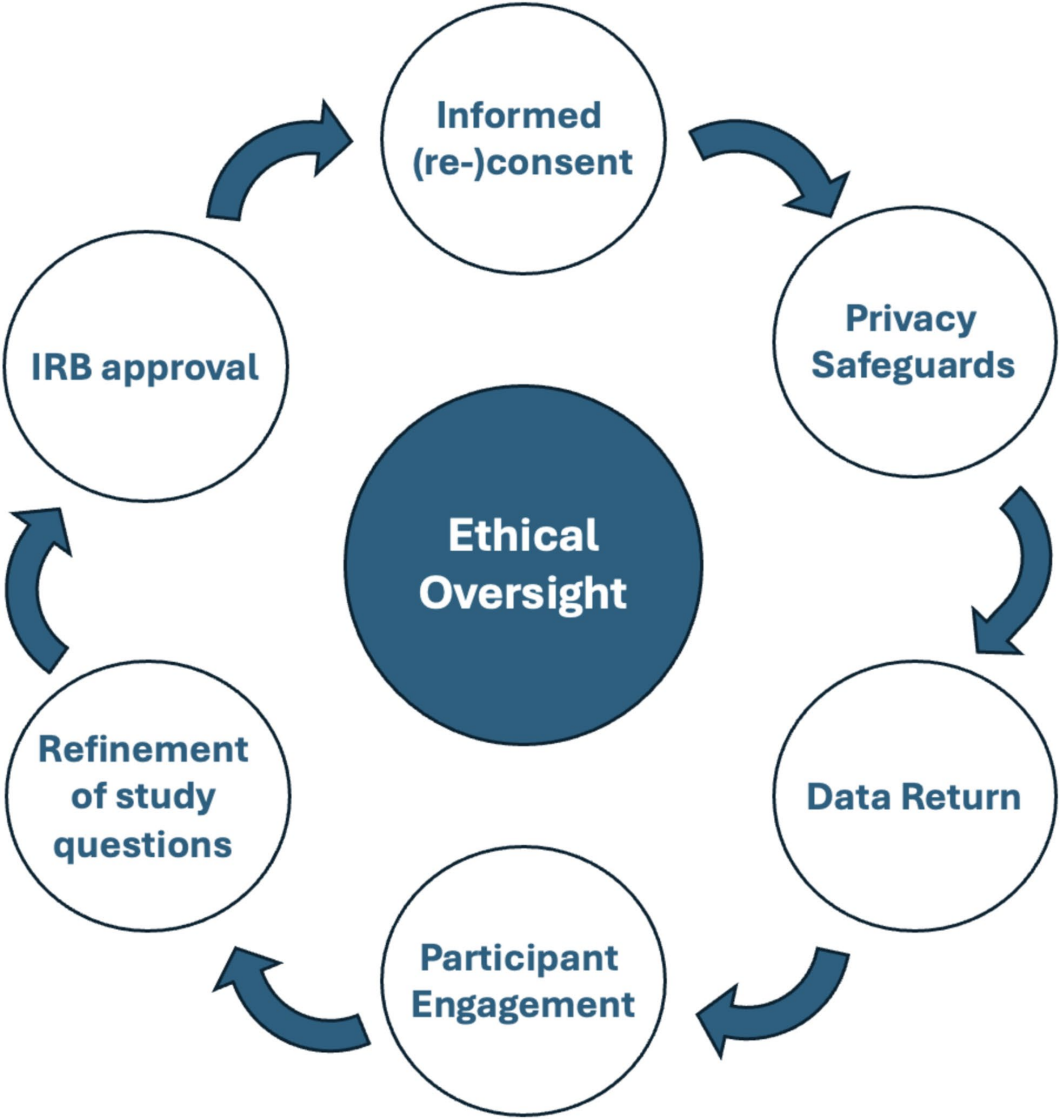
Ethical guidance cycle for exposome research. This schematic illustrates core elements of ethical oversight including informed consent, re-consent, privacy safeguards, data return policies, IRB approval, and participant engagement

Just as the Human Genome Project catalyzed the institutionalization of ELSI research, the Human Exposome Project must invest in a new ethical infrastructure (Fig. 4): one that reflects its multisystemic scope, vulnerability to commercialization, and embeddedness in socio-ecological injustice. An Exposome Ethics Consortium, supported by public agencies, could develop guidelines, coordinate stakeholder forums, and ensure that the ethics of exposome research evolves alongside its methods and technologies.

To enhance context and conceptual coherence, this revised version integrates programmatic insights and conceptual frameworks from early exposome ethics literature. Foundational initiatives—such as HELIX [71], HEALS,^13^ EXPOsOMICS,^14^ EXIMIOUS,^15^ CHEAR [72], NEUROSOME,^16^ URBANOME,^17^ NEXUS,^18^ HHEAR2, IHEN^19^, and ENVESOME^20^—are now referenced to highlight the evolution of ethical practices in large-scale environmental health studies.^21^ The European Human Exposome Network (EHEN)1, particularly its Working Group on Ethics and Law, is noted for advancing participatory ethics and regulatory alignment within the EU.

The future of exposomics hinges not only on scientific innovation but on ethical imagination. If the exposome is to serve as the foundation of a new public health paradigm, it must be built on ethical principles that extend beyond the lab and clinic into the neighborhoods, lives, and futures of those most affected by environmental harm. Therefore, it formed a central part of the 2025 Human Exposome Moonshot Forum.

In addition, ethics infrastructure should incorporate Evidence-to-Decision (EtD) frameworks [73], enabling transparent, participatory translation of exposomic data into policy. These frameworks should evaluate evidence quality, benefit-risk balance, feasibility, equity, and stakeholder values, ensuring exposomic knowledge leads to action.

## Validation ethics and evidence-to-decision framework in exposomics

**Fig. 5:**
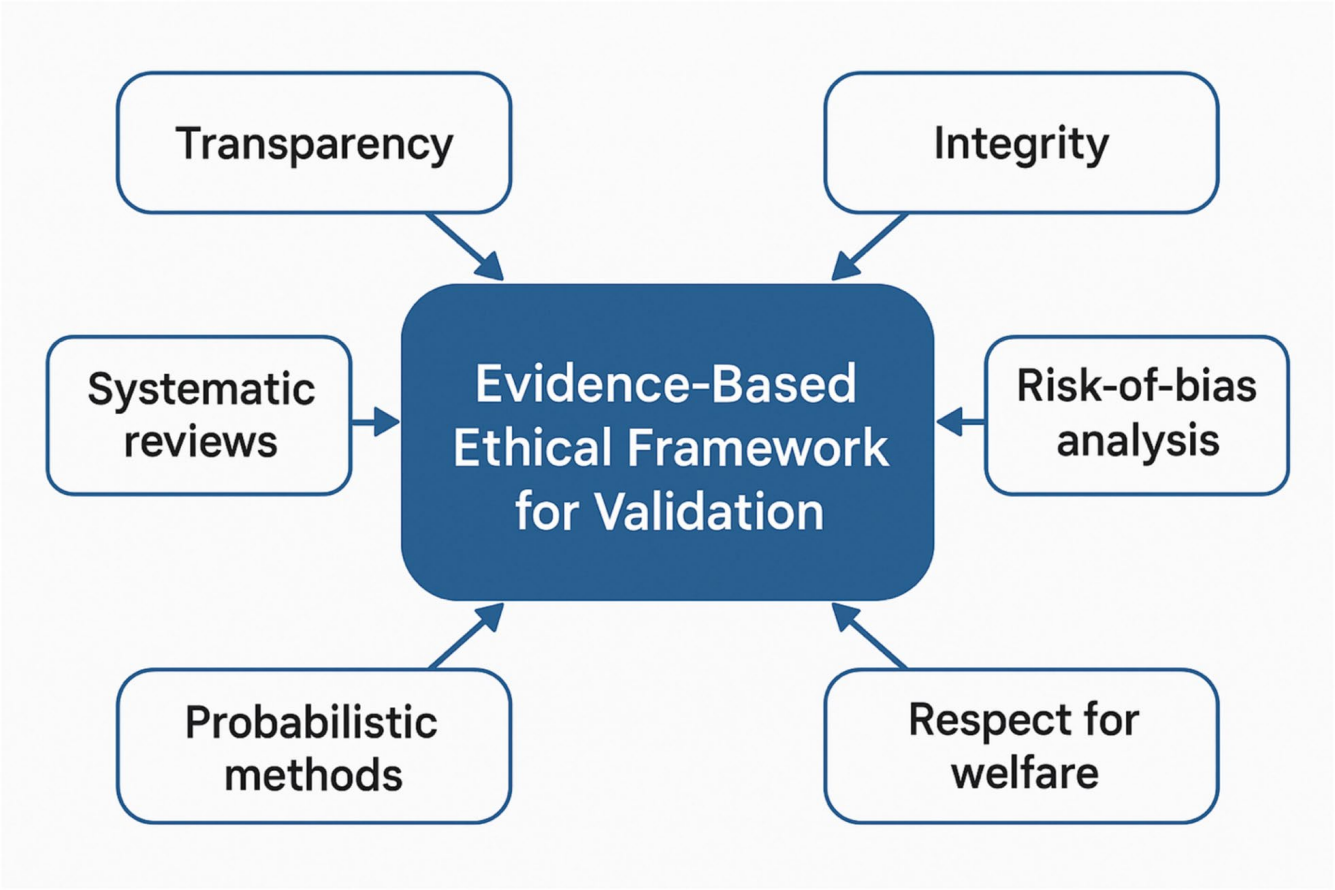
Evidence-based ethical framework for validation (adapted from Hartung [64]). Core components include transparency, integrity, risk-of-bias analysis, systematic reviews, probabilistic methods, and respect for welfare, supporting the validation of complex, non-animal-based exposomic methods. Validation of exposomic tools cannot rely on outdated templates centered on animal concordance. As Hartung [64] argues, an ethical validation framework must prioritize human relevance, flexibility, and transparency, embracing a probabilistic, contextualized, and iterative model

Validation in exposomics is not simply a technical exercise: it is a normative act. It determines whose data count, which risks are prioritized, and how precautionary action is justified. The many challenges in validating methods, exposure hypotheses, and conclusions require the application of Validation Ethics [64]. A dedicated ethics framework for validation is essential in exposome research, where tools often lack clear precedents in animal models and operate under high uncertainty. Hartung proposes reframing validation as a moral and epistemic exercise grounded in human relevance, probabilistic reasoning, and systematic evaluation (Fig. 5). The ethical principles include transparency, integrity, risk-of-bias control, evidence synthesis, and respect for human and animal welfare. These principles form the basis of Evidence-Based Toxicology (EBT)^23^ [74], which aligns well with the goals of exposomics to generate actionable, population-level insights.

In particular, the Evidence-to-Decision (EtD) framework currently developed in EBTC provides a systematic roadmap for translating complex exposome data into actionable decisions. While EBT aims to come to the best evidence, EtD aims to come to the best resulting advice for action. It is essentially a structured decision matrix that bridges research findings to real-world applications, ensuring that insights from exposomics (e.g., patterns of lifetime environmental exposures) are prioritized and interpreted with policy in mind [75]. Crucially, this approach makes the rationale behind decisions explicit and transparent, which is invaluable for risk communication—stakeholders can see why certain exposure risks are deemed a priority and understand the evidence behind recommendations. By laying out decision criteria in advance, the EtD framework also facilitates public health translation of exposome research, so that scientific findings on cumulative exposures are more readily turned into concrete interventions or guidelines. At the same time, regulators can integrate exposome data more confidently, since the framework compels consideration of feasibility and context before new rules are set, leading to decisions that are both evidence-based and broadly accepted [76, 77]. In short, an EtD-guided process helps ensure that exposomics findings inform policies in a way that is objective, transparent, and responsive to public values.

### Structure of the EtD framework

The EtD approach proceeds through a series of domains or criteria that collectively guide ethical and policy-relevant interpretation of evidence.^24^ It begins with careful problem formulation, defining the exposure–health question and decision context (e.g., identifying which environmental exposure or mixture is of concern, in which population and setting). Next comes evidence synthesis, which involves assembling and appraising the relevant exposomic evidence—often large-scale epidemiological data, biomonitoring results, or mechanistic findings—and judging its certainty or quality. With the problem and evidence base established, the framework then directs attention to key considerations: the balance of benefits and harms (weighing the desirable health benefits of addressing an exposure against any potential adverse effects or unintended consequences), values and preferences (how much affected communities, decision-makers or other stakeholders value the outcomes in question and what trade-offs they are willing to accept), resource use (the cost and resource implications of any action, such as implementing an exposure mitigation program or further monitoring, and whether those resources are justified by the expected benefit), equity (whether the decision would promote health equity), feasibility (the practical likelihood that a proposed policy or intervention can be successfully implemented, given technical, logistical or political constraints), and acceptability (the degree of support or opposition likely from the public, industry, and policymakers for the proposed action). By systematically evaluating each of these domains, the EtD framework ensures that decisions on exposome-related risks are comprehensive and ethical: not only is the scientific evidence considered, but so are societal values, practical trade-offs, and fairness. This multi-criteria structure guards against a narrow focus on hazard alone, prompting decision-makers to explicitly justify how they move from exposome science to recommendations.

The EtD should specify who pays and through what mechanism, requiring a payer‑specific budget‑impact analysis alongside cost‑effectiveness and affordability evidence tailored to the implementing setting [78]. Where exposures arise along product and supply chains, apply polluter‑pays and extended producer‑responsibility principles so monitoring and mitigation costs are not shifted onto exposed communities; in low‑resource contexts, align with international initiatives (e.g., the Global Framework on Chemicals) to mobilize capacity and support.^25^ To safeguard equity, pair the economic appraisal with distributional cost‑effectiveness analysis and report the costs of inaction to justify investment and prioritize fair burden‑sharing [79].

### Guiding risk communication, public health action, and regulation

Applying the EtD framework in exposomics yields several tangible benefits for communication and governance. First, the transparency of the process directly improves risk communication [73]. When authorities use an EtD table or narrative, they can clearly communicate why a certain environmental risk was prioritized and how scientific evidence underpins the decision. For example, if a community is concerned about a mixture of air pollutants, an EtD analysis would document the decision reasoning (e.g., showing that the health benefits of reducing those pollutants outweigh harms, that the community values cleaner air, and that a mitigation plan is feasible and cost-effective). Such clarity helps build public trust and understanding, as people see their values and concerns explicitly factored into the outcome. Second, the framework’s emphasis on context and feasibility aids public health translation of exposome data. Rather than simply identifying statistically significant exposure-disease links, EtD forces a discussion on how to act on that information—be it through targeted interventions, health advisories, or policy changes. This means exposomics evidence is more likely to lead to real public health measures, because decision-makers have walked through the practical steps and resource needs in advance. Third, for regulatory integration, the EtD criteria align closely with what regulators must consider when setting standards or guidelines. Regulators are tasked not just with assessing hazards, but with balancing costs, technical feasibility, stakeholder acceptance, and equity, all elements built into the EtD approach. By using an EtD framework, regulatory bodies can justify decisions (such as new exposure limits or product restrictions) in a way that is systematic and defensible, showing they have evaluated all relevant factors and stakeholder viewpoints. Notably, recent adaptations of GRADE/EtD for environmental health emphasize socio-political context and accommodating diverse stakeholder values, which speaks to the ethical imperative of inclusivity in exposome governance.

## Conclusion: from reactive to proactive ethics

As the HEP accelerates into a new era of data-driven, sensor-enabled, and AI-integrated science, it must not inherit the reactive ethical posture that has often trailed behind genomic, digital health, and environmental surveillance innovations. Instead, exposomics offers a rare opportunity to place ethics *ahead* of the curve to treat normative reflection not as an afterthought or compliance checkbox, but as a constitutive part of scientific design, governance, and translation.

*Ethical leadership must precede, not follow, technological innovation.* As exposome tools become more precise and pervasive, ethical oversight must evolve in parallel to ensure that these tools do not entrench social inequalities, commodify lived experience, or render individuals and communities vulnerable to surveillance and exploitation [8, 69]. We must anticipate harms and co-create safeguards, particularly in contexts where data, once collected, can rarely be recalled, and where the power to interpret and act on such data is unequally distributed across stakeholders.

*The Human Exposome Project must be built on robust ethical scaffolding that fosters public trust, global equity, and scientific excellence.* Just as the HGP catalyzed the ELSI program and reshaped public engagement with genetics, the exposome requires its own ethical infrastructure: one that is transdisciplinary, reflexive, and responsive to both individual and collective concerns. This infrastructure should include embedded ethics researchers, community advisory mechanisms, and regulatory innovations such as exposome-specific data governance protocols and algorithmic audit tools [9, 26].

The ethical leadership required for the HEP must be institutionalized. We call for the dedicated funding and establishment of the *International Exposome Ethics Consortium (EEC)* outlined above. It should be analogous to the ELSI network but tailored to the specific challenges of environmental, social, and biological complexity—it should be a priority for funders and policymakers worldwide [21, 70]. This body, funded by national and international health research agencies and governed by a diverse multi-stakeholder board, is essential to provide the proactive, specialized guidance that local IRBs cannot. Its mandate will be to develop anticipatory guidelines, train the next generation of researchers and reviewers, and ensure that ethical standards evolve in lockstep with exposomic methods. By investing in this ethical scaffolding, we can ensure that the HEP's legacy is not only one of scientific discovery but also of a more just and equitable model for large-scale, data-intensive health research.

*Ethical leadership is not merely about avoiding scandal or ensuring compliance. It is about envisioning and building a future in which science serves society justly, transparently, and inclusively*. The HEP, in seeking to uncover the environmental determinants of health, holds immense promise for transforming preventive medicine and population health [80]. But its legitimacy, sustainability, and transformative power will depend not only on scientific rigor, but on ethical foresight. Its true legacy will depend on whether it also redefines how ethics is practiced in large-scale, data-intensive science.

We emphasize that the frameworks and typologies presented here are starting points, i.e., conceptual tools derived from ethical analysis rather than co-produced consensus. Their refinement through participatory engagement with affected communities, regulators, and policymakers represents an essential next phase in building a truly inclusive exposome ethics infrastructure.

As HEP accelerates into a new era of AI-enabled, sensor-integrated, and globally distributed research, its legitimacy hinges on ethical scaffolding that ensures scientific utility, social inclusion, and public benefit. Through an institutionalized Exposome Ethics Consortium, participatory governance models, dynamic consent systems, and context-aware result communication, we can build a HEP that advances environmental health without reinforcing injustice. Ethics is not aconstraint but a generative force—one that guides the Human Exposome Project toward just, transparent, and inclusive futures.

## Data Availability

No datasets were generated or analyzed during the current study.

## Abbreviations

AI: Artificial Intelligence
ATHLETE: Advancing Tools for Human Early‑Life Exposome Research and Translation (EU Horizon 2020 project)
BPA: Bisphenol A
CAB: Community Advisory Board
CBPR: Community‑Based Participatory Research
DTC: Direct‑to‑Consumer (testing/services)
EBT: Evidence‑Based Toxicology
EHEN: European Human Exposome Network
ELSI: Ethical, Legal and Social Implications
ENVESOME: European Human ENVironment ExposOME projetc (EU Horizon 2020 project)
EtD: Evidence‑to‑Decision (framework)
EXIMIOUS: Exposome‑Impacted Immunological Outcomes in Human Populations (EU Horizon 2020 project)
EXPOsOMICS: Integrated Exposure Science of Exposomics (EU FP7 project)
GDPR: General Data Protection Regulation (EU)
HEALS: Health and Environment—Analysis for Life Sciences (EU FP7 project)
HEP: Human Exposome Project
HELIX: Human Early-Life Exposome Project
HGP: Human Genome Project
HHEAR: Human Health Exposure Analysis Resource (US NIH project)
HIPAA: Health Insurance Portability and Accountability Act (US)
IRB: Institutional Review Board
NEUROSOME: Neurodevelopmental Exposome project (EU Horizon 2020 project)
NEXUS: Network for EXposomics in the United States (US NIH project)
SHAP: SHapley Additive exPlanations (xAI technique)
URBANOME: URBAN Observatory for Multi‑dimensional Exposome (EU Horizon 2020 project)
xAI: Explainable Artificial Intelligence

## Footnotes

1 https://www.humanexposome.eu.

2 https://hhearprogram.org.

3 https://athleteproject.eu.

4 https://exposomemoonshot.org.

5 https://factor.niehs.nih.gov/2025/6/feature/global-exposome-consortium.

6 https://exposomemoonshot.org.

7 https://www.genome.gov/Funded-Programs-Projects/ELSI-Research-Program-ethical-legal-social-implications.

8 https://www.eeoc.gov/statutes/genetic-information-nondiscrimination-act-2008.

9 https://www.gfbr.global/wp-content/uploads/2025/04/GFBR-2025-background-paper-FINAL.pdf.

10 https://www.go-fair.org/fair-principles/.

11 https://unesdoc.unesco.org/ark:/48223/pf0000381137.

12 https://www.who.int/publications/i/item/9789240029200.

13 http://www.heals-eu.eu/index.php/project/.

14 https://exposomics-project.cc.ic.ac.uk/live-exposome.pantheonsite.io/who-are-we.html.

15 https://www.eximious-h2020.eu.

16 https://www.neurosome.eu.

17 https://www.urbanome.eu.

18 https://www.nexus-exposomics.org.

19 https://humanexposome.net.

20 https://cordis.europa.eu/project/id/101157269.

21 https://humanexposomeproject.com.

23 https://www.ebtox.org.

24 https://www.medwave.cl/revisiones/metodinvestreport/8182.html?_view=en.

25 https://www.oecd.org/en/publications/the-polluter-pays-principle_9789264044845-en.html.

## LLM use statement

Various AI models based on Large Language Models were used for literature search, supporting English usage by non-native speakers, and checking compliance with journals' reporting standards. Generative AI (ChatGPT-5) was used to create icons for graphics. The authors take full responsibility for the final product.
